# Circ-ADAM9 targeting PTEN and ATG7 promotes autophagy and apoptosis of diabetic endothelial progenitor cells by sponging mir-20a-5p

**DOI:** 10.1038/s41419-020-02745-x

**Published:** 2020-07-13

**Authors:** Ding Tian, Yin Xiang, Yong Tang, Zhuowang Ge, Qianhui Li, Yachen Zhang

**Affiliations:** Department of Cardiology, Xinhua Hospital, Shanghai Jiaotong University School of Medicine, Shanghai, China

**Keywords:** miRNAs, Peripheral vascular disease

## Abstract

Dysfunction of endothelial progenitor cells (EPCs) is a key factor in vascular complications of diabetes mellitus. Although the roles of microRNAs and circular RNAs in regulating cell functions have been thoroughly studied, their role in regulating autophagy and apoptosis of EPCs remains to be elucidated. This study investigated the roles of mir-20a-5p and its predicted target circ-ADAM9 in EPCs treated with high glucose (30 mM) and in a diabetic mouse hind limb ischemia model. It is found that Mir-20a-5p inhibited autophagy and apoptosis of EPCs induced by high-concentration glucose. Further, mir-20a-5p could inhibit the expression of PTEN and ATG7 in EPCs, and promote the phosphorylation of AKT and mTOR proteins under high-glucose condition. Investigation of the underlying mechanism revealed that circ-ADAM9, as a miRNA sponges of mir-20a-5p, promoted autophagy and apoptosis of EPCs induced by high-concentration glucose. Circ-ADAM9 upregulated PTEN and ATG7 in interaction with mir-20a-5p, and inhibited the phosphorylation of AKT and mTOR to aggravate autophagy and apoptosis of EPCs under high glucose. In addition, silencing of circ-ADAM9 increased microvessel formation in the hind limbs of diabetic mice. Our findings disclose a novel autophagy/apoptosis-regulatory pathway that is composed of mir-20a-5p, circ-ADAM9, PTEN, and ATG7. Circ-ADAM9 is a potential novel target for regulating the function of diabetic EPCs and angiogenesis.

## Introduction

Diabetes mellitus is a group of metabolic diseases characterized by hyperglycemia. Diabetic macro- and microvascular complications are the main causes of death and disability in patients with diabetes mellitus, and endothelial dysfunction is one of the major causes of diabetic vascular complications^1^. Endothelial progenitor cells (EPCs) can differentiate into endothelial cells and/or promote angiogenesis, which has a key role in the repair of endothelial dysfunction^2,3^. A reduction in EPCs can lead to vascular endothelial dysfunction, as has been observed in patients with diabetes^4^. Thus, exploring new molecules that can prevent the hyperglycemia-induced damage of EPCs is of great significance for the treatment of diabetes mellitus.

Autophagy and apoptosis are important regulatory processes of intracellular homeostasis. Autophagy is an evolutionarily conserved stress response^5,6^. It is generally considered a protective mechanism because it provides nutrition for cells and eliminates damaged organelles. However, autophagy can also lead to excessive consumption of intracellular proteins and organelles as well as the degradation of anti-apoptotic and cell-survival factors, resulting in autophagic cell death and apoptosis^7–9^. Apoptosis is a process in which cells stop growing and dividing and enter into a controlled cell death without affecting the surrounding cell environment. To further investigate autophagy and apoptosis of EPCs in diabetic pathological environment is an effective way to explore the functional changes of diabetic EPCs.

MicroRNAs (miRNAs) can negatively regulate gene expression by inhibiting RNA translation or promoting RNA degradation^10^. MiRNAs have key roles in regulating autophagy, apoptosis, and angiogenesis of diabetic EPC^11–14^. The polycistronic miRNA cluster mir-17–92, including mir-20a, has proven roles in regulating the function of vascular endothelial cells and promoting angiogenesis^15–18^. However, the role of mir-20a in EPC injury induced by high glucose has not been revealed.

Circular RNAs (circRNAs) are a type of non-coding RNAs in which the 3′ and 5′ ends are joined together, forming a closed continuous loop^19^. They are produced through back-splicing of a pre-mRNA in which a splicing donor is joined to an upstream splicing acceptor. Because of their closed-loop structure, circRNAs are not easily degraded by RNase R and are more stable than linear RNAs^20^. More than 10,000 circRNAs have been identified, and they have been widely studied because of their unique structure, conservativeness across species, cell-type and tissue specificity, and stable expression in saliva, blood, and exosomes^21–23^. Although the functions of circRNAs have not been completely revealed, it is generally believed that they can act as miRNA sponges to regulate gene expression and may have potential in diagnosis and treatment^24^.

Inspired by the above findings, we speculated that circRNAs might sponge miRNAs and thus interfere with EPCs to regulate their biological functions in the diabetic environment. In this study, we exposed EPCs to high-concentration glucose in vitro to investigate the effect and mechanism of circRNAs binding with mir-20a-5p on EPC function and angiogenesis under high-glucose condition. The regulatory role of circRNAs on angiogenesis in vivo was investigated in a diabetic hind limb ischemia mouse model. The results showed that circ-ADAM9 (has_circ_0001791) acts as a mir-20a-5p sponge to regulate autophagy and apoptosis of EPCs, thus regulating angiogenesis in diabetic pathological conditions. Circ-ADAM9 represents a potential new treatment target for diabetic angiopathy.

## Results

### Identification of human umbilical vein blood-derived EPCs

EPCs were derived from human umbilical cord blood. Immunostaining and flow cytometry were used to detect the expression of cell-specific antigens in adherent colonies. The umbilical vein-derived cells stained strongly positive for the endothelial lineage markers CD31 and VEGFR-2 (Supplementary Fig. S1A, C). CD34 and CD133 were also expressed in these cells (Supplementary Fig. S1A, C). In addition, the cells were able to absorb ac-LDL, bind to UEA-1, and form capillary-like structures on Matrigel (Supplementary Fig. S1B, D), which are characteristics of endothelial lineage cells^25^. These findings clearly indicated that we successfully isolated EPCs from human umbilical vein blood.

### Mir-20a-5p regulates apoptosis and autophagy of EPCs induced by high glucose in vitro

**Fig. 1:**
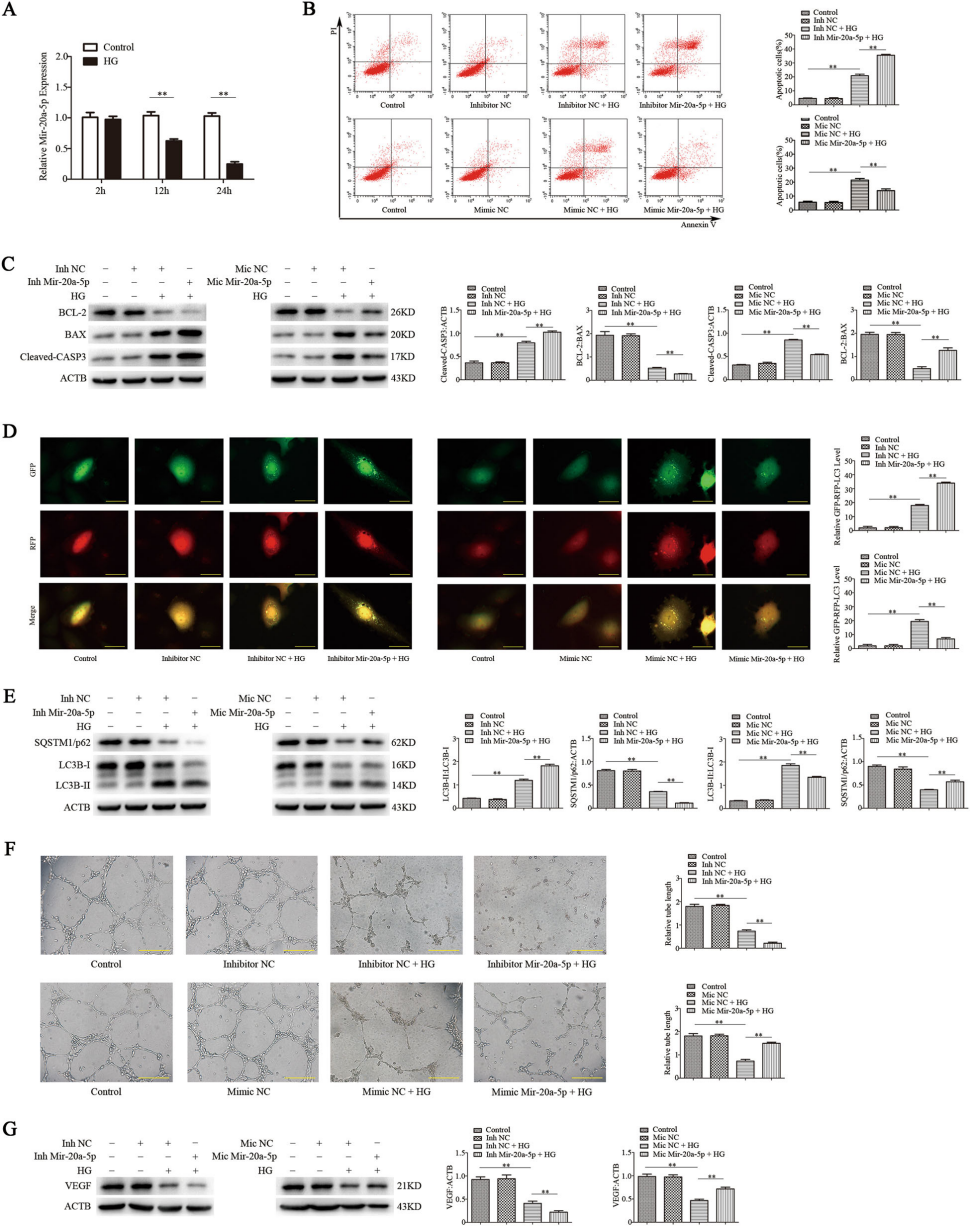
Mir-20a-5p regulates apoptosis, autophagy, and angiogenic function of EPCs induced by high glucose in vitro. EPC dysfunction was induced by high glucose (30 mM) treatment for 24 h. Mannitol was used as an osmolar control treatment. EPCs were transfected with mir-20a-5p or negative control (NC) inhibitor, or mir-20a-5p or NC mimic. **a** RT-qPCR analysis of mir-20a-5p expression. **b** Flow-cytometric analysis of apoptosis using annexin V/propidium iodide (PI). Annexin V^+^/PI^+^ or Annexin V^+^/PI^−^ (quadrants 2 and 3) cells were defined as apoptotic cells. **c** Protein levels of BCL-2, BAX, and cleaved-CASP3 as detected by western blotting. **d** Representative images showing LC3 staining in different groups of EPCs infected with GFP-RFP-LC3 adenovirus for 24 h. Scale bar: 20 μm. **e** Western blot analysis of LC3B-II/LC3B-I and SQSTM1/p62 levels. **f** The angiogenic capability of EPCs was determined by a tube formation assay. Tube length was normalized to that in the control group. Scale: 200 μm. **g** Protein levels of VEGF as detected by western blotting. Inh inhibito, Mic mimic, *P < 0.05, **P < 0.01, *n* = 3.

We first examined whether mir-20a-5p affects the function of EPCs under high glucose. EPCs were cultured in vitro in high-glucose (30 mM) medium for 24 h to mimic the diabetic condition. An RT-qPCR assay revealed that high-glucose treatment significantly decreased the level of mir-20a-5p in a time-dependent manner (Fig. 1a). Next, we overexpressed or knocked down mir-20a-5p in EPCs. Flow-cytometric analysis revealed that overexpression of mir-20a-5p significantly reduced the number of apoptotic cells induced by high glucose, whereas knockdown of mir-20a-5p further promoted the apoptosis of EPCs under high-glucose stimulation (Fig. 1b). Western blot analysis showed that mir-20a-5p overexpression reduced the expression of BAX and cleaved-CASP3 under high-glucose condition, whereas mir-20a-5p knockdown had the opposite effects (Fig. 1c). Thus, mir-20a-5p can directly suppress the apoptosis of EPCs induced by high glucose. RFP-GFP-LC3B analysis of the autophagic flux showed that mir-20a-5p overexpression reversed the autophagy of EPCs induced by high glucose, and mir-20a-5p knockdown further promoted autophagy (Fig. 1d). Western blot analysis showed that overexpression of mir-20a-5p inhibited the upregulation of LC3B-II under high glucose and increased the level of SQSTM1/p62. In contrast, mir-20a-5p knockdown enhanced the expression of LC3B-II under high glucose (Fig. 1e). These findings suggested that mir-20a-5p suppresses autophagy of EPCs under high-glucose condition.

### Mir-20a-5p regulates the angiogenic function of EPCs induced by high glucose in vitro

We used tube formation assays to measure the angiogenic function of EPCs. Overexpression of mir-20a-5p inhibited the negative effect of hyperglycemia on the tubulogenic capacity of EPCs, whereas knockdown of mir-20a-5p further reduced the tubulogenic capacity of EPCs (Fig. 1f). Western blot analysis also showed that overexpression of mir-20a-5p could reverse the decrease of vascular endothelial growth factor (VEGF) expression in EPCs under high-glucose condition, whereas mir-20a-5p knockdown had the opposite effects (Fig. 1g). These findings indicated that mir-20a-5p enhances the angiogenic function of EPCs under high-glucose condition.

### PTEN and ATG7 are direct targets of mir-20a-5p in EPCs

**Fig. 2:**
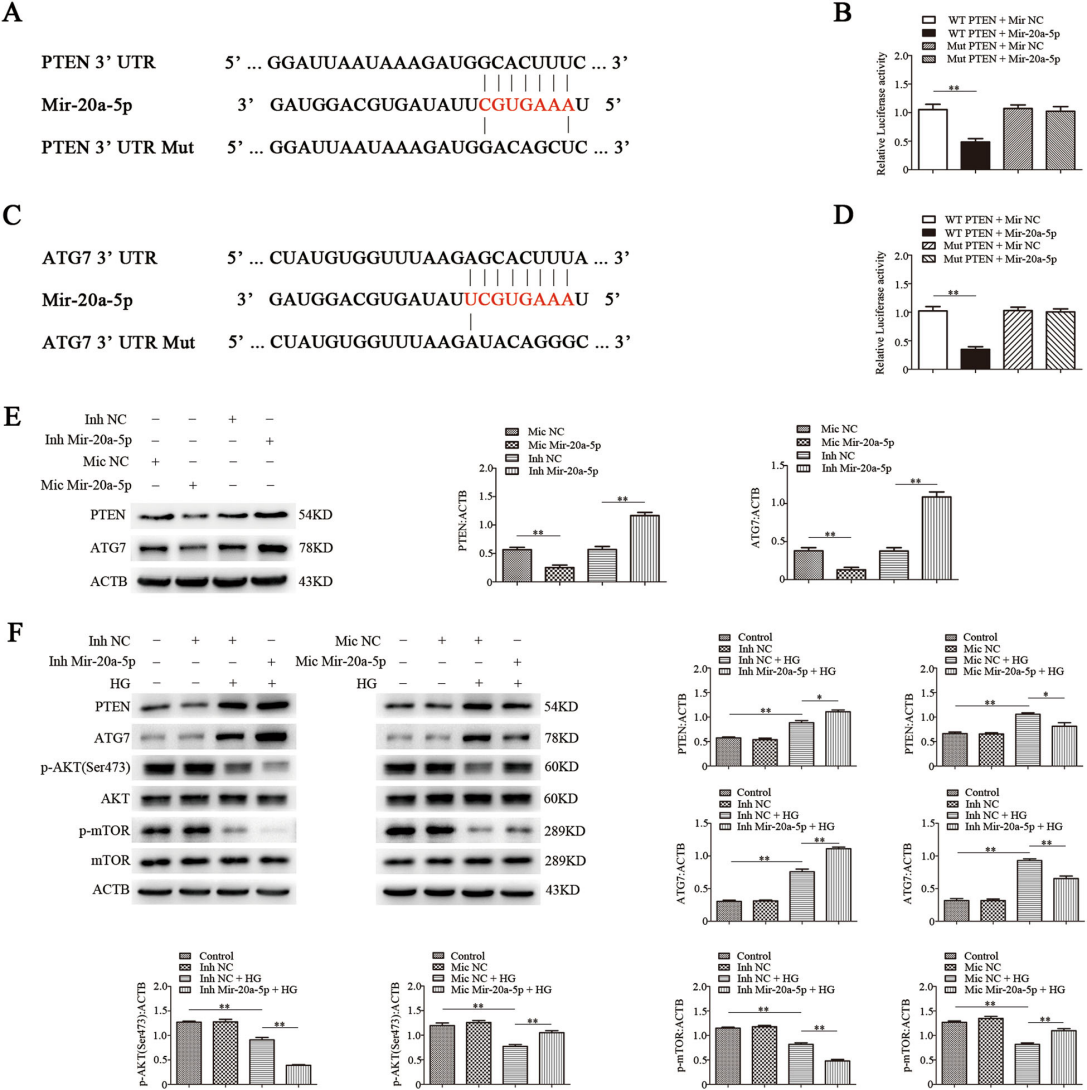
PTEN and ATG7 are direct targets of mir‑20a‑5p, and mir-20a-5p regulates PTEN, ATG7, and AKT/mTOR signaling in EPCs induced by high glucose. **a**, **c** Schematic representation of the putative target sites of mir-20a-5p in the 3′-UTRs of ATG7 and PTEN, and the sequence of mir-20a-5p mutant (indicated as Mut). **b**, **d** Luciferase assay of cells cotransfected with wild-type (WT) or mutated (Mut) ATG7 3′-UTR or PTEN 3′-UTR reporter construct and mir-20a-5p or the control for 48 h. **e** Western blot analysis of PTEN and ATG7 protein levels in EPCs treated with mir-20a-5p inhibitor or mimics. **f** Western blot analysis of PTEN, ATG7, p-AKT, and p-mTOR protein levels in EPCs treated with mir-20a-5p inhibitor or mimics under high-glucose condition. Inh inhibitor, Mic mimic, *P < 0.05, **P < 0.01, *n* = 3.

mRNA targets of mir-20a-5p were identified by bioinformatics analysis using TargetScan. We found that mir-20a-5p targets and regulates the translation of PTEN and ATG7. To confirm the prediction, we conducted luciferase reporter assays after cotransfection of EPCs with wild-type (WT) or mutated (Mut) ATG7 3′-UTR or PTEN 3′-UTR (Fig. 2a, c) and mir-20a-5p mimics or controls. Mir-20a-5p suppressed the luciferase activity in cells transfected with ATG7 3′-UTR or PTEN 3′-UTR, but not in cells transfected with Mut-ATG7 3′-UTR or Mut-PTEN 3′-UTR (Fig. 2b, d). These results suggested that mir-20a-5p negatively regulates the expression of both genes by directly binding their 3′-UTR. Western blot analysis corroborated that PTEN and ATG7 are direct targets of mir-20a-5p in EPCs. Knockdown of mir-20a-5p significantly increased the expression of PTEN and ATG7, whereas overexpression of mir-20a-5p suppressed their expression (Fig. 2e).

### Mir-20a-5p regulates the PTEN, ATG7, and AKT/mTOR signaling pathways in EPCs induced by high glucose

PTEN activates autophagy through the AKT/mTOR pathway to control cellular energy homeostasis^26,27^. *ATG7*, as an autophagy-related gene, has an important role in autophagy^28^. To verify the regulatory effects of mir-20a-5p on PTEN and ATG7 in high glucose-induced EPCs, we exposed EPCs with overexpression or knockdown of mir-20a-5p to high glucose or to mannitol as an osmolar control treatment. Western blot analysis showed that PTEN and ATG7 levels were significantly increased after high-glucose treatment, whereas and AKT and mTOR phosphorylation was significantly reduced. Overexpression of mir-20a-5p reversed the high expression of PTEN and ATG7 under high glucose and increased the phosphorylation levels of AKT and mTOR. In contrast, knockdown of mir-20a-5p further increased PTEN and ATG7 expression under high-glucose condition and significantly reduced the phosphorylation levels of AKT and mTOR (Fig. 2f).

### Circ-ADAM9 directly sponges mir‑20a‑5p in EPCs

**Fig. 3:**
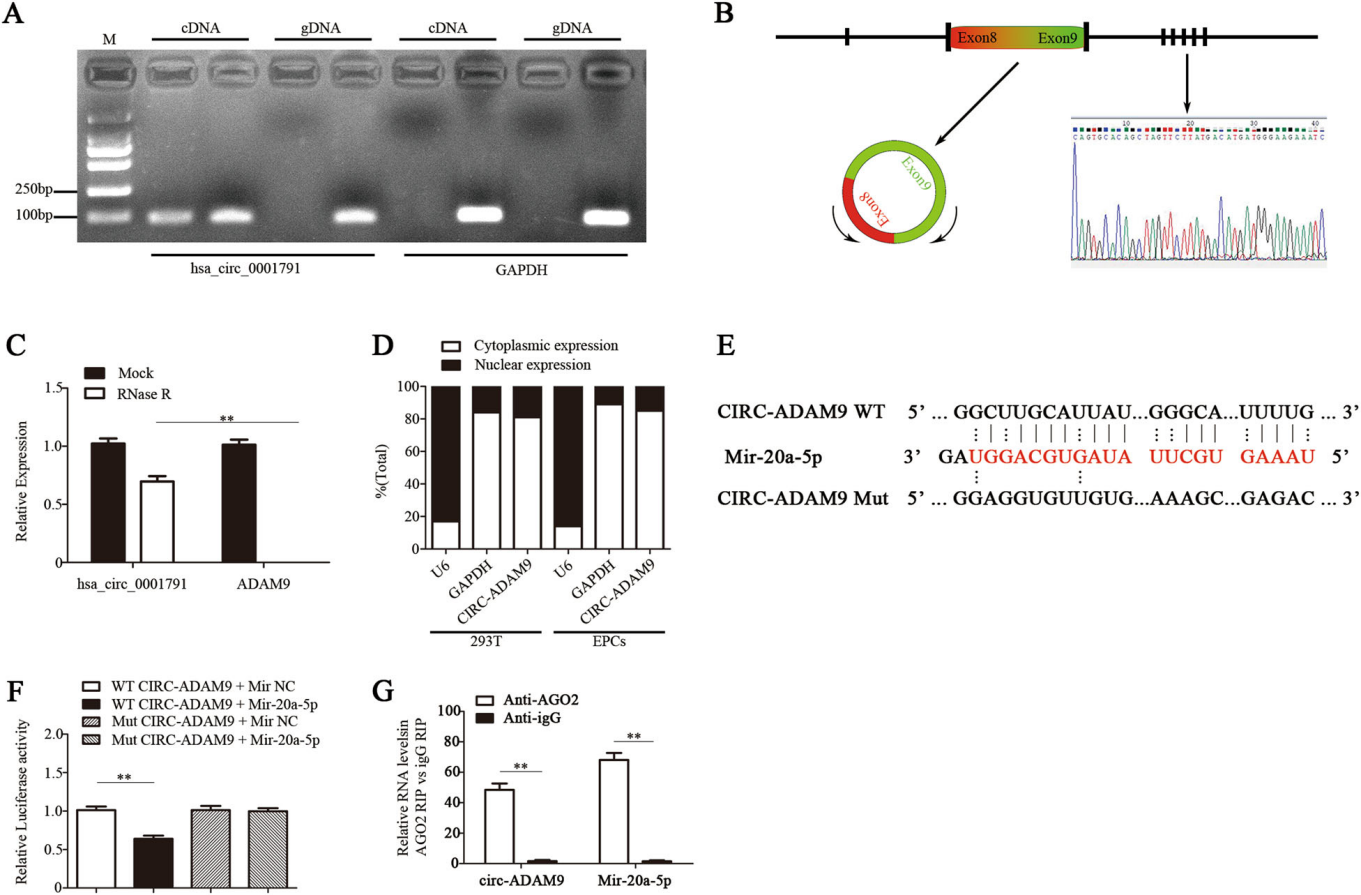
Circ-ADAM9 directly sponges mir‑20a‑5p in EPCs. **a** Gel electrophoresis was used to show the expression of circ-ADAM9 and linear GAPDH transcript in cDNA and gDNA. **b** Schematic illustration showing ADAM9 exon 8 and exon 9 circularization forming circ-ADAM9. The presence of circ-ADAM9 was validated by RT-qPCR followed by Sanger sequencing. **c** PCR analysis confirmed that linear ADAM9 could be easily digested by RNase R, whereas circ-ADAM9 resisted to RNase R digestion. **d** Levels of nuclear control transcript (U6), cytoplasmic control transcript (GAPDH), and circ-ADAM9 in nuclear and cytoplasmic fractions as assessed by RT-qPCR. **e** Schematic illustration of the complementarity of the mir-20a-5p seed sequence with circ-ADAM9. **f** EPCs were cotransfected with mir-20a-5p mimics or control and a luciferase reporter construct containing wild-type (WT) or mutated (Mut) circ-ADAM9. **g** RIP assay to verify whether circ-ADAM9 and mir-20a-5p directly bind to AGO2 in EPCs. *P < 0.05, **P < 0.01, *n* = 3.

To identify circRNAs that might act as a sponge of mir-20a-5p in EPCs, we screened 112 circRNAs based on the prediction of circRNA recognition elements in the mir-20a-5p sequence by each of miRanda, TargetScan, and RNAhybrid (Supplementary Fig. S2A). CircRNAs have been shown to regulate the expression of their host genes, and share some similar functions^29,30^. Therefore, we selected 15 circRNAs whose host genes are closely related to vascular function from these 112 circRNAs. RT-qPCR analysis showed that among these candidates, circ-ADAM9 (has_circ_0001791) was significantly increased in EPCs under high-glucose condition (30 mM for 24 h) (Supplementary Fig. S2B). Considering its abundance and sequence length, we speculated that circ-ADAM9 might have a key role in the functional regulation of diabetic EPCs as a competing endogenous RNA by binding mir-20a-5p. CircBase retrieval showed that circ-ADAM9 (hsa_circ_0001791) is located at chr8:38879161–38880844 in the human genome. Gel electrophoresis showed that circ-ADAM9 is highly expressed in EPCs. The gene was amplified from cDNA only when using divergent primers, whereas linear *GAPDH* transcripts could be amplified from both cDNA and gDNA using convergent primers (Fig. 3a). Next, we confirmed head-to-tail splicing in the circ-ADAM9 RT-qPCR product and the circ-ADAM9 size by Sanger sequencing (Fig. 3b). Further, we used RNase R to determine the expression of circ-ADAM9 and linear ADAM9 by RT-qPCR in EPCs. The results showed that circ-ADAM9 is obviously resistant to RNase R, whereas the expression of linear ADAM9 was significantly decreased after RNase R treatment (Fig. 3c). To identify the cellular localization of circ-ADAM9, cells were separated into nuclear and cytoplasmic fractions. U6 mainly exists in the nucleus, whereas GAPDH exists only in the cytoplasm. Circ-ADAM9 was mainly located in the cytoplasmic fraction of 293T cells and EPCs cells (Fig. 3d), which means that circ-ADAM9 might have a regulatory role after transcription. Bioinformatics prediction revealed that mir-20a-5p is a common miRNA that potentially binds to circ-ADAM9 (Fig. 3e). Upon transfection of EPCs with mir-20a-5p mimics or controls, compared with that of the Mut reporter, the luciferase activity of the WT reporter was significantly reduced (Fig. 3f). The AGO2 protein is a core component of the RNA-induced silencing complex. A RIP assay revealed that circ-ADAM9 and mir-20a-5p were especially abundant in the immunoprecipitate pulled down with anti-AGO2, but not in that of anti-IgG (Fig. 3g). These results suggested that circ-ADAM9 functions as mir-20a-5p sponge.

### Circ-ADAM9 regulates apoptosis and autophagy of EPCs induced by high glucose in vitro

**Fig. 4:**
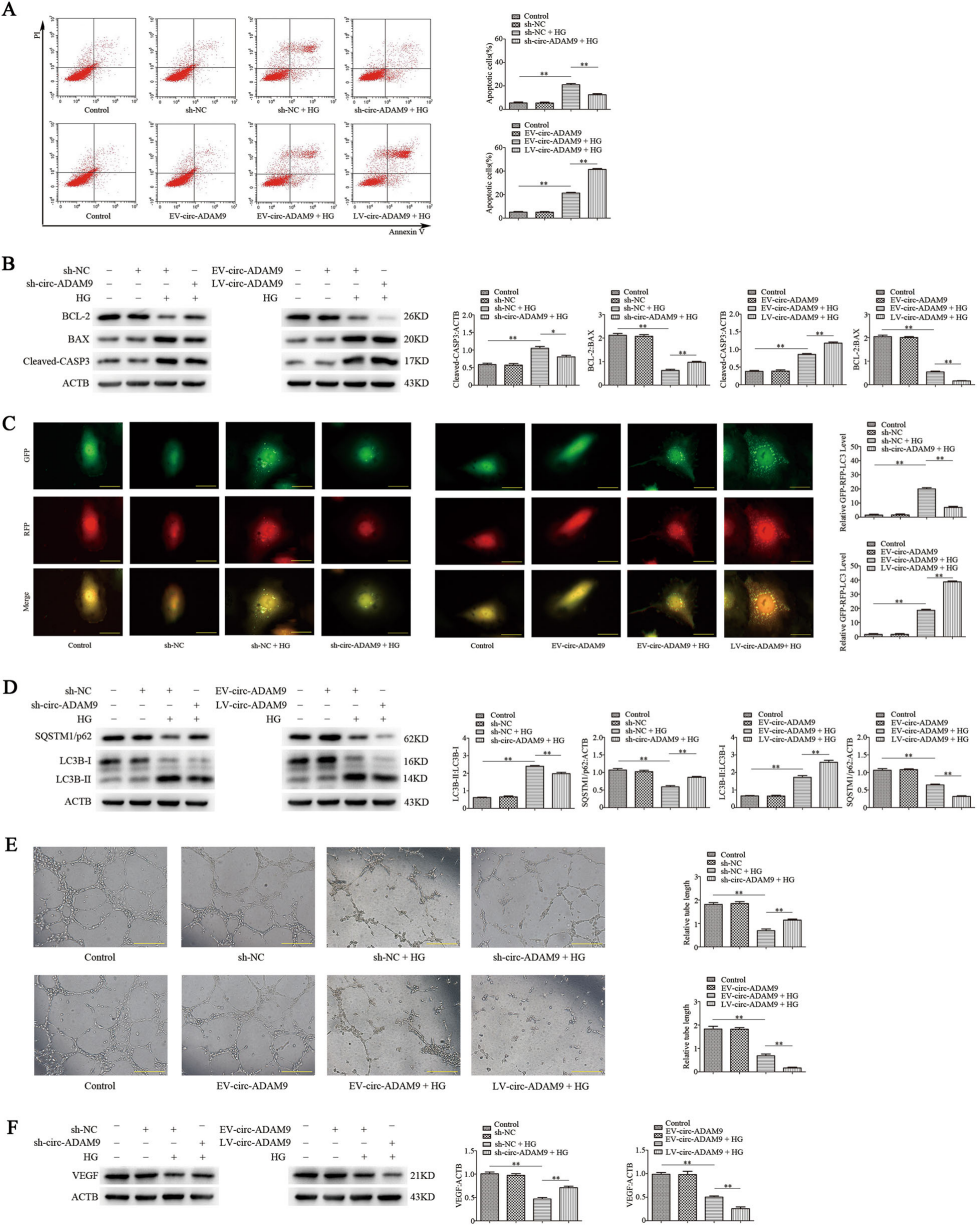
Circ-ADAM9 regulates apoptosis, autophagy, and angiogenic function of EPCs induced by high glucose. EPC dysfunction was induced by high glucose (30 mM) treatment for 24 h. Mannitol was used as an osmolar control treatment. Cells were transfected with circ-ADAM9 knockout (sh-circ-ADAM9) or overexpression (LV-circ-ADAM9) lentiviral construct or negative control lentivirus (sh-NC or EV-circ-ADAM9). **a** Flow-cytometric analysis of apoptosis using annexin V/PI. Annexin V^+^/PI^+^ or Annexin V^+^/PI^–^ (quadrants 2 and 3) cells were defined as apoptotic cells. **b** Protein levels of cleaved-CASP3, BAX, and BCL-2 as detected by western blotting. **c** Representative images showing LC3 staining in different groups of EPCs infected with GFP-RFP-LC3 adenovirus for 24 h. Scale bar: 20 μm. **d** Western blot analysis of LC3B-II/LC3B-I and SQSTM1/p62 levels. **e** The angiogenic capability of EPCs was determined by a tube formation assay. Tube length was normalized to that in the control group. Scale: 200 μm. **f** Protein levels of VEGF as detected by western blotting. *P < 0.05, **P < 0.01, *n* = 3.

To confirm the regulatory effect of circ-ADAM9 on EPCs under high glucose, we overexpressed and knocked down circ-ADAM9 in EPCs by lentivirus transfection, and induced EPC dysfunction by exposure to high glucose (30 mM) for 24 h. Flow-cytometric analysis revealed that compared with non-stimulated EPCs, the number of apoptotic cells increased significantly after high-glucose stimulation, whereas knockdown of circ-ADAM9 significantly suppressed apoptosis induced by high glucose. Overexpression of circ-ADAM9 further promoted EPC apoptosis under high-glucose stimulation (Fig. 4a). Western blot analysis showed that knockdown of circ-ADAM9 reduced the expression of BAX and cleaved-CASP3 under high-glucose condition, whereas circ-ADAM9 overexpression had the opposite effects (Fig. 4b). Thus, circ-ADAM9 promotes apoptosis of EPCs induced by high glucose. RFP-GFP-LC3B analysis revealed that the autophagic flux of EPCs stimulated with high glucose was significantly higher than that of non-stimulated EPCs, whereas autophagy was suppressed and further enhanced upon knockdown and overexpression of circ-ADAM9, respectively (Fig. 4c). Western blot analysis indicated that knockdown of circ-ADAM9 inhibited the upregulation of LC3B-II under high glucose and increased the level of SQSTM1/p62. In contrast, overexpression of circ-ADAM9 enhanced the expression of LC3B-II under high glucose (Fig. 4d). These findings indicated that circ-ADAM9 enhances autophagy of EPCs under high-glucose condition.

### Circ-ADAM9 regulates the angiogenic function of EPCs induced by high glucose in vitro

Knockdown of circ-ADAM9 inhibited the negative effect of hyperglycemia on the tubulogenic capacity of EPCs, whereas overexpression of circ-ADAM9 further weakened their tubulogenic capacity (Fig. 4e). Western blot analysis showed that over knockdown of circ-ADAM9 could reverse the decrease of VEGF expression in EPCs under high-glucose condition, whereas overexpression of circ-ADAM9 had the opposite effects (Fig. 4f). These findings suggested that circ-ADAM9 suppresses angiogenic function of EPCs under high-glucose condition.

### Circ-ADAM9 regulates autophagy and apoptosis of EPCs under high-glucose condition through mir-20a-5p

**Fig. 5:**
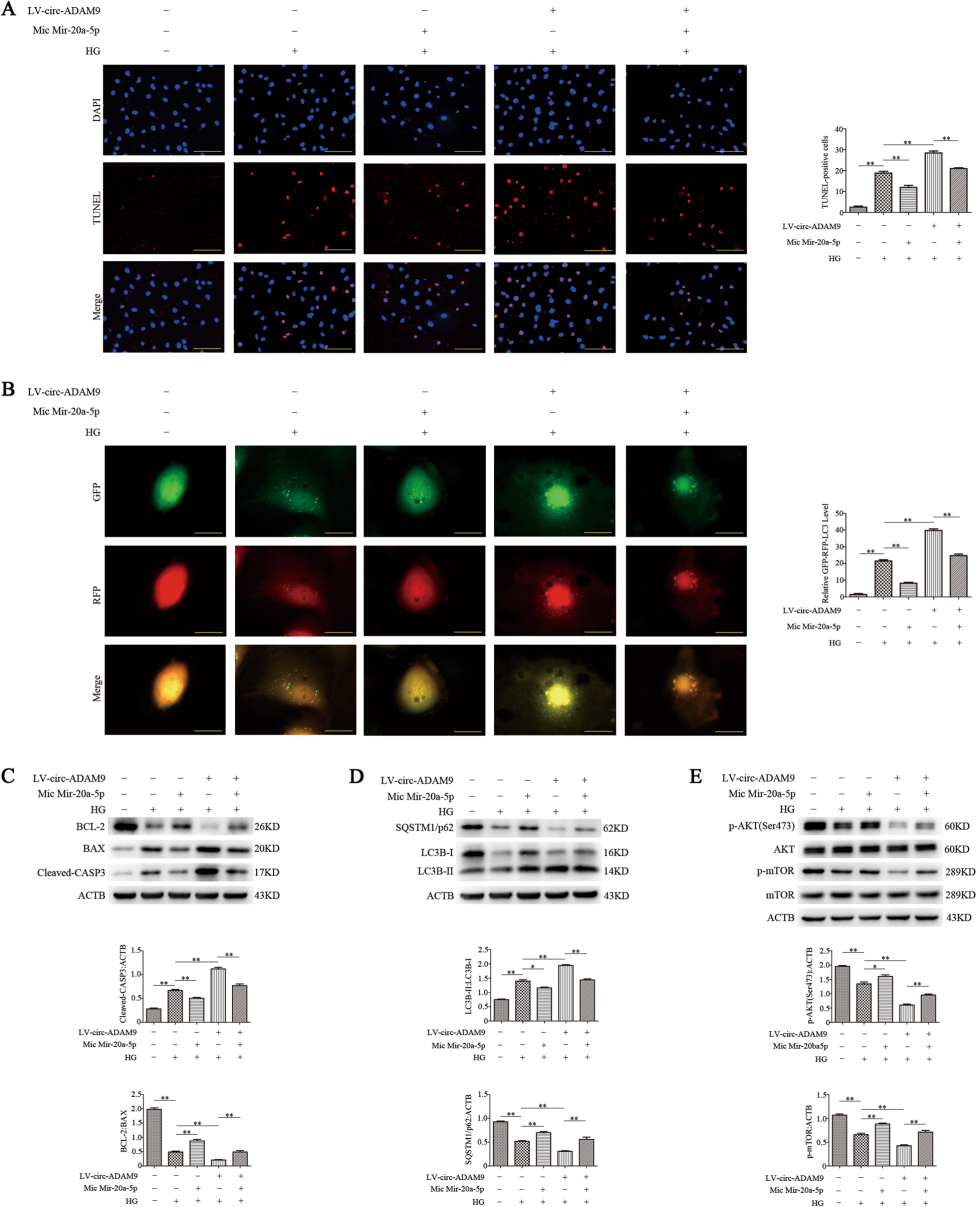
Circ-ADAM9 directly binds to mir-20a-5p and regulates the expression of AKT and mTOR proteins, thereby regulating the autophagy and apoptosis of EPCs under high-glucose condition. EPC dysfunction was induced by high glucose (30 mM) treatment for 24 h. The cells were transfected with mir-20a-5p mimic, LV-circ-ADAM9, or mir-20a-5p mimic + LV-circ-ADAM9 before the high glucose treatment. **a** TUNEL staining of apoptotic cells. Scale: 100 μm. **b** Representative images of LC3 staining in different groups of EPCs infected with GFP-RFP-LC3 adenovirus for 24 h. Scale bar: 20 μm. **c** Protein levels of BCL-2, BAX, and cleaved-CASP3 as detected by western blotting. **d** Western blot analysis of LC3B-II/LC3B-II and SQSTM1/p62 levels. **e** Levels of p-AKT and p-mTOR as detected by western blotting. *P < 0.05; **P < 0.01; *n* = 3.

The above findings indicated that circ-ADAM9 can interact with mir-20a-5p, and circ-ADAM9 can directly regulate autophagy and apoptosis of EPCs under high-glucose condition. To confirm whether circ-ADAM9 regulates autophagy and apoptosis of EPCs through mir-20a-5p, we simultaneously overexpressed circ-ADAM9 and mir-20a-5p in EPCs and then exposed the cells to high glucose. Mir-20a-5p inhibited the increases in EPC autophagy and apoptosis induced by overexpression of circ-ADAM9 under high-glucose condition (Fig. 5a, b). Western blot analysis revealed that mir-20a-5p reversed the upregulation of cleaved-CASP3, BAX, and LC3B-II, and the downregulation of SQSTM1/p62, p-AKT, and p-mTOR by circ-ADAM9 under high glucose (Fig. 5c–e). These results suggested that circ-ADAM9 regulates the expression of AKT and mTOR proteins by directly binding to mir-20a-5p, thereby regulating autophagy and apoptosis of EPCs under high-glucose condition.

### Circ-ADAM9 regulates autophagy and apoptosis of EPCs under high-glucose condition by targeting PTEN and ATG7

**Fig. 6:**
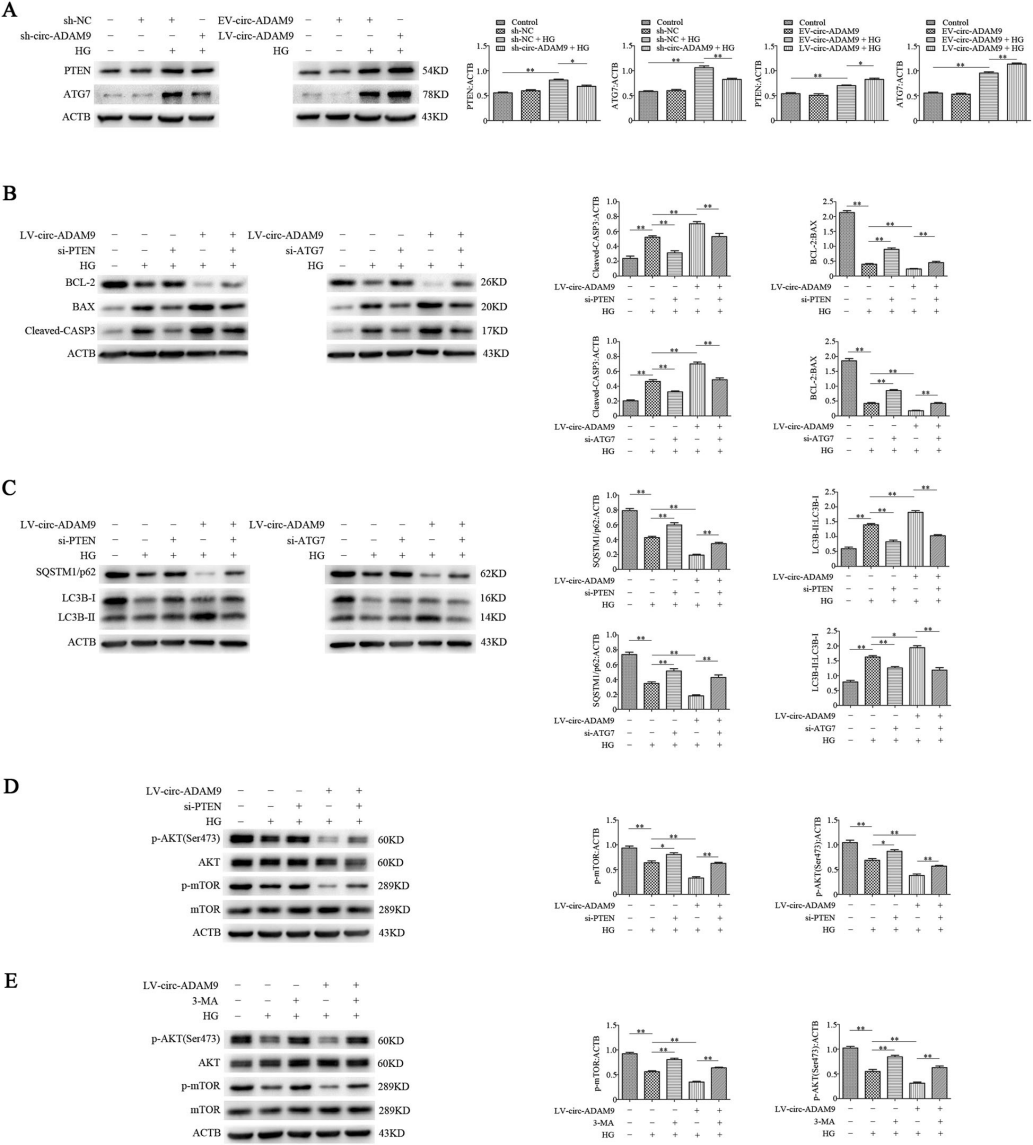
Circ-ADAM9 targets PTEN/AKT/mTOR and ATG7 to regulate autophagy and apoptosis of EPCs under high-glucose condition. **a** Western blot analysis of PTEN and ATG7 protein levels in EPCs under high-glucose condition upon overexpression and knockdown of circ-ADAM9. **b** Western blot analysis of BCL-2, BAX, and cleaved-CASP3 protein levels in cells transfected with si-PTEN/si-ATG7 and LV-circ-ADAM9. **c** Western blot analysis of LC3B-II/LC3B-II and SQSTM1/p62 levels in cells transfected with si-PTEN/si-ATG7 and LV-circ-ADAM9. **d** Western blot analysis of p-AKT and p-mTOR levels after transfection with si-PTEN and LV-circ-ADAM9. **e** Western blot analysis of p-AKT and p-mTOR levels after transfection with 3-MA and LV-circ-ADAM9. *P < 0.05; **P < 0.01; *n* = 3.

To evaluate whether circ-ADAM9 targets PTEN and ATG7 in EPCs, we used EPCs with overexpression or knockdown of circ-ADAM9 and exposed them to high glucose or mannitol as an osmolar control. Western blot analysis revealed that knockdown of circ-ADAM9 reversed the high expression of PTEN and ATG7 under high glucose. Conversely, overexpression of circ-ADAM9 further increased their expression under high glucose (Fig. 6a). To determine whether circ-ADAM9 regulates autophagy and apoptosis of EPCs by targeting PTEN and ATG7, we overexpressed circ-ADAM9 and knocked down PTEN and ATG7 (Supplementary Fig. S3) in EPCs, which were then exposed to high glucose. Knockdown of PTEN and ATG7 reversed the upregulation of cleaved-CASP3, BAX, and LC3B-II, and the downregulation of BCL-2, SQSTM1/p62 induced by circ-ADAM9 under high glucose (Fig. 6b, c). Western blot analysis revealed that knockdown of PTEN as well as treatment with the autophagy inhibitor 3-methyladenine (3-MA) counteracted the inhibitory effect of circ-ADAM9 on p-AKT and p-mTOR levels (Fig. 6d, e). These results indicated that circ-ADAM9 targets PTEN-regulated AKT/mTOR signaling and directly targets the autophagy-related gene *ATG7* to regulate autophagy and apoptosis of EPCs under high-glucose condition.

### Downregulation of circ-ADAM9 in EPCs promotes angiogenesis in hind limbs of diabetic mice

**Fig. 7:**
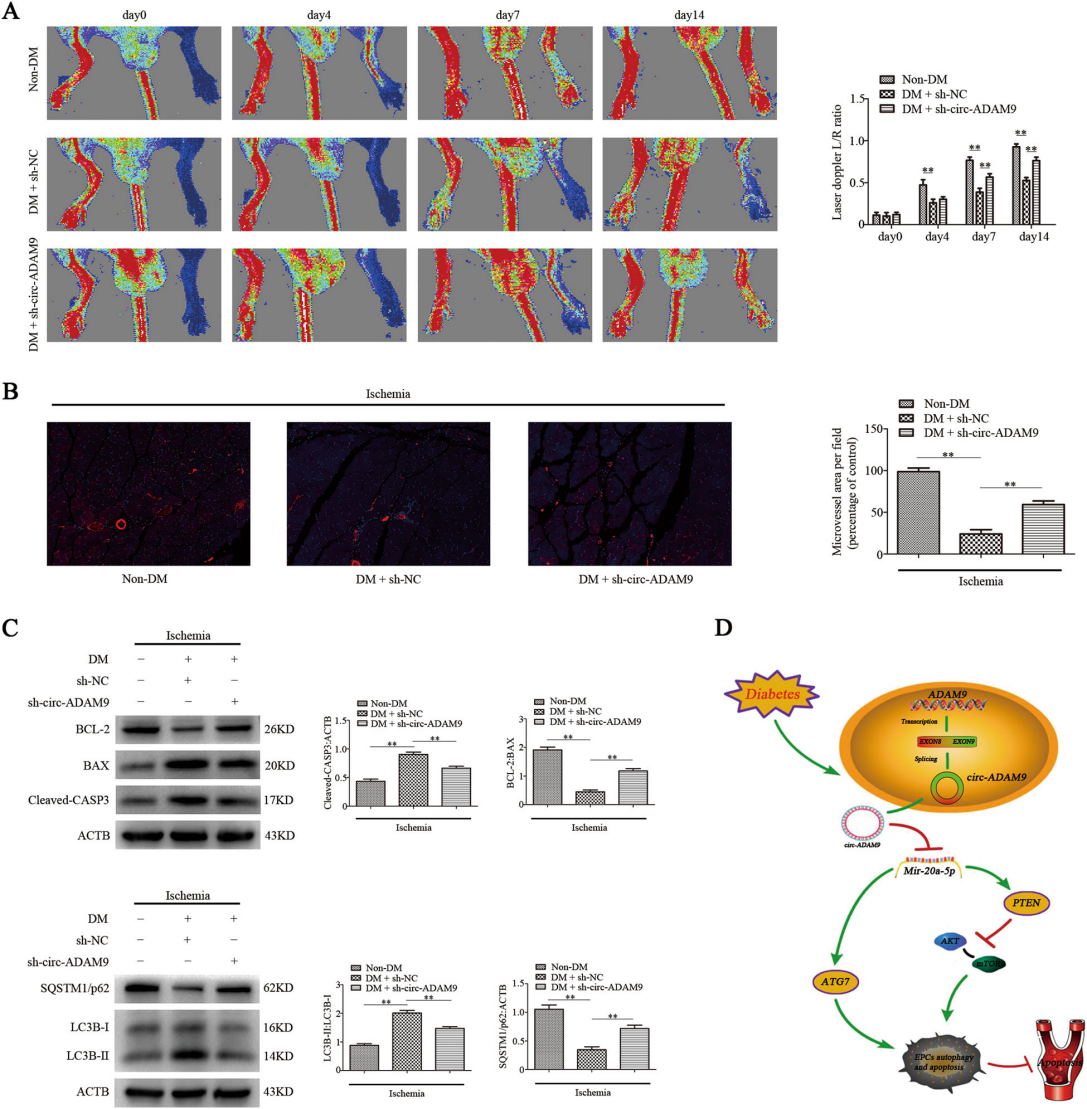
Downregulation of circ-ADAM9 in EPCs promotes angiogenesis in hind limbs of diabetic mice. **a** Non-diabetic and diabetic nude mice with hind limb ischemia were injected with human umbilical vein EPCs transfected with lentivirus sh-circ-ADAM9or sh-NC. Blood flow in hind limbs was measured using a laser Doppler imaging system on days 0, 4, 7, and 14 after injection. Representative images and blood flow ratios are shown. **b** CD31-specific immunofluorescence staining (red) in ischemic muscle tissues of nude mice 14 days after injection of the cells. **c** Western blot analysis of BCL-2, BAX, cleaved-CASP3, LC3B-II/LC3B-II, and SQSTM1/p62 levels. **d** Circ-ADAM9 promotes the expression of PTEN and decreases the phosphorylation of AKT/mTOR, and circ-ADAM9 directly induces the expression of the autophagy-related gene *ATG7*, leading to the enhancement of autophagy and apoptosis of EPCs in diabetes. *P < 0.05, **P < 0.01; *n* = 6.

To evaluate whether knockdown of circ-ADAM9 can effectively improve the apoptosis of EPCs induced by high glucose and enhance their angiogenesis, we established a diabetic nude mouse model of hind limb ischemia. Human umbilical vein EPCs with knockdown of circ-ADAM9 (sh-circ-ADAM9) or control cells (sh-NC) were injected intramuscularly at six sites on the ischemic limb distal to the vascular occlusion site. Laser Doppler measurements showed that compared with non-diabetic nude mice (non-DM), the recovery of blood perfusion in the hind limbs of diabetic nude mice was significantly slower, whereas it was significantly quicker in diabetic nude mice injected with circ-ADAM9 human umbilical vein EPCs than in mice in the lentiviral control group on days 7 and 14 (Fig. 7a). Ischemic muscle tissues of mice in each group were collected 7 days after the injection of human umbilical vein EPCs for immunofluorescence staining of CD31, an endothelial cell marker. Compared with non-DM mice, diabetic nude mice showed significantly reduced CD31 staining in ischemic muscle tissue. CD31 staining in ischemic muscle tissues of diabetic nude mice injected with circ-ADAM9-knockdown human umbilical vein EPCs was significantly increased compared with that in the lentiviral control group (Fig. 7b). At the same time, western blot analysis also showed that compared with non-diabetic nude mice, autophagy and apoptosis-related protein expression of LC3B, cleaved-CASP3, BAX in ischemic muscle tissue of diabetic nude mice increased significantly, while SQSTM1/p62 and BCL-2 decreased. However, the expression of protein in ischemic muscle of diabetic nude mice injected with circ-ADAM9-knockout EPCs were reversed (Fig. 7c). These results suggested that knockdown of circ-ADAM9 in diabetic human EPCs rescues their angiogenic ability and reduce autophagy and apoptosis in vivo.

## Discussion

EPCs have a key role in the repair of endothelial dysfunction by secreting a variety of growth factors or differentiating into mature endothelial cells. Previous studies have shown that the pathological environment of diabetes can lead to the decrease of EPCs and the impairment of EPCs function^31–33^. How to avoid the functional damage of EPCs is significant for the prevention of diabetic vascular complications.

Autophagy and apoptosis are both important physiological processes affecting cell function. Apoptosis can promote cell damage and affect cell survival. Autophagy, under some circumstances, may increase apoptosis and cell damage, and reduce the ability of angiogenesis^34–37^, since excessive autophagy can lead to abnormal consumption of intracellular proteins and organelles, increase of degradation of anti-apoptotic and cell-survival factors, resulting in autophagic cell death and apoptosis^7–9^. Our preliminary study confirmed that autophagy and apoptosis of EPCs increase significantly under high glucose conditions, and suppress angiogenesis. On this basis, we look forward to further exploring new molecules and their underlying mechanisms of autophagy and apoptosis of EPCs in high glucose environment.

MiRNAs, as a non-coding RNA, have an important role in regulating the apoptosis, autophagy and angiogenesis of EPCs under diabetic pathological environment^11–14^. Our further findings confirmed mir-20a-5p inhibited autophagy and apoptosis of EPCs induced by high glucose, and promoted the angiogenic function of EPCs. It is well known that PTEN can activate autophagy to control the cellular energy balance through the AKT/mTOR pathway^26,27^. mTOR is a highly conserved serine/threonine protein kinase that exists in two complexes: mTORC1 and mTORC2. AKT can directly phosphorylate pras40, activate the mTORC1 pathway, and regulate cell autophagy, proliferation, and growth^38,39^. ATG7 has an important role in autophagy^28^. Mir-20a reportedly regulates PTEN and ATG7 expression, participates in glycogen synthesis in hepatocytes, and inhibits autophagy of macrophages^40,41^. Our study revealed that mir-20a-5p could inhibit the expression of PTEN and ATG7 in EPCs at the transcriptional and translational levels. Further, mir-20a-5p can reverse the high expression of PTEN and ATG7 and promote the phosphorylation of AKT and mTOR proteins under high-glucose condition.

CircRNAs are pivotal in gene expression regulation at the post-transcriptional level. CircRNAs can act as competing endogenous RNAs and the crosstalk between circRNAs, miRNAs, and targets represents a complex gene expression-regulatory network^42^. It has been found that circRNAs can affect vascular endothelial function and regulate angiogenesis by sponging miRNAs^43,44^. In this study, we found a circRNA, circ-ADAM9, which is extensively expressed in EPCs and binds and sponges mir-20a-5p. In contrast to mir-20a-5p, circ-ADAM9 was found to promote autophagy and apoptosis of EPCs induced by high glucose and to inhibit the angiogenic function of EPCs.

We also found that the increase in autophagy and apoptosis of EPCs induced by overexpression of circ-ADAM9 was partially offset upon simultaneous overexpression of mir-20a-5p. In addition, in contrast to the effect of mir-20a-5p, overexpression of circ-ADAM9 inhibited AKT and mTOR phosphorylation in EPCs under high glucose, whereas this inhibitory effect was weakened upon simultaneous overexpression of circ-ADAM9 and mir-20a-5p. This suggests that circ-ADAM9 promotes autophagy and apoptosis of EPCs under high glucose through interacting with mir-20a-5p and participating in the regulation of AKT/mTOR signaling. We also found that, in contrast to mir-20a-5p, circ-ADAM9 promoted the expression of PTEN and ATG7 under high-glucose condition. When circ-ADAM9 was overexpressed and PTEN was knocked down in EPCs under high glucose, the increases in autophagy- and apoptosis-related protein expression induced by overexpression of circ-ADAM9 and the decrease in AKT and mTOR phosphorylation were reversed. This confirmed that circ-ADAM9 regulates autophagy and apoptosis of EPCs through the PTEN/AKT/mTOR pathway. Meanwhile, knockout of ATG7 counteracted the increases in autophagy- and apoptosis-related protein expression induced by overexpression of circ-ADAM9 under high glucose, indicating that circ-ADAM9 can directly act on ATG7, leading to autophagy and apoptosis of EPCs under high-glucose condition. Further, we found that treatment with the autophagy inhibitor 3-MA rescued the reduction in AKT and mTOR phosphorylation in EPCs after overexpression of circ-ADAM9. These results indicate that circ-ADAM9 directly binds to mir-20a-5p to regulate autophagy and apoptosis of EPCs via the PTEN/AKT/mTOR pathway and ATG7. Finally, we verified that knockdown of circ-ADAM9 can reduce autophagy and apoptosis, and improve angiogenesis under diabetic conditions in vivo.

In summary, binding of circ-ADAM9 with mir-20a-5p promotes autophagy and apoptosis of EPCs under high-glucose condition. On the one hand, circ-ADAM9 promotes PTEN expression and suppresses AKT/mTOR phosphorylation, which aggravates autophagy and apoptosis of EPCs under high-glucose condition. On the other hand, circ-ADAM9 directly induces the expression of the autophagy-related gene *ATG7*, which also leads to the enhancement of autophagy and apoptosis of EPCs under high glucose. Therefore, circ-ADAM9 may be a new therapeutic target for the prevention of angiogenesis impairment induced by dysfunction of diabetic EPCs.

## Materials and methods

### EPC culture, identification, and treatment

EPCs were derived from human umbilical cord blood. We obtained informed consent from the parents of the infants, and this study was approved by the Institutional Review Board at Xinhua Hospital, Shanghai Jiaotong University School of Medicine. EPCs were extracted, cultured, and identified as described previously^45^. Cells were exposed to 30 mM glucose as a high-glucose treatment, and mannitol was used as an osmolar control.

### RNA isolation and quantitative reverse-transcription (RT-q) PCR analysis

Total RNA was extracted using TRIzol reagent (cat. no. 15596018; Invitrogen). Cytoplasmic and nuclear fractions were prepared using an NE-PER extraction kit (Pierce). CircRNAs, miRNAs, and mRNAs were quantified by qPCR using specific primers (Table 1) and a SYBR Premix Ex Taq™ kit (TaKaRa, Dalian, China). *GAPDH* and *U6* were amplified as internal controls. Relative expression was calculated using the comparative threshold cycle method^46^.

**Table 1:** PCR primers used in this work.

Gene symbol	Polarity	Sequence
mir-20a-5p	Forward	5′-TAAAGTGCTTATAGTGCAGGTAG-3′
	Reverse	Uni-miR qPCR primer (Takara, Dalian, China)
PTEN	Forward	5′-TGGATTCGACTTAGACTTGACCT-3′
	Reverse	5′-GCGGTGTCATAATGTCTCTCAG-3′
ATG7	Forward	5′-TGCTATCCTGCCCTCTGTCTT-3′
	Reverse	5′-TGCCTCCTTTCTGGTTCTTTT-3′
hsa_circ_0003550	Forward	5′-GGGAAGCGATGATAACTGCG-3′
	Reverse	5′-GCAATGTAGAATCCGCTGGG-3′
hsa_circ_0005441	Forward	5′-AGTGTCCGTACGATCCCAAG-3′
	Reverse	5′-GGAGGCTGCCCAGTGTATAA-3′
hsa_circ_0012671	Forward	5′-TGAGACACTACTACCACCGC-3′
	Reverse	5′-AAGCAGTGCAGATGTGTTGG-3′
hsa_circ_0037936	Forward	5′-ACTCTCCTTGCAGTCCAGTC-3′
	Reverse	5′-ATCCATTGTGTTCCAGCCAC-3′
hsa_circ_0011128	Forward	5′-CAATACCATCGCCATGCACA-3′
	Reverse	5′-CCACTGCCAGGTTGTCTACT-3′
hsa_circ_0003973	Forward	5′-TGACCACCAGATGAACCACA-3′
	Reverse	5′-CGAACTCACACAGCTCCATG-3′
hsa_circ_0027772	Forward	5′-TGCTGCCCATTATACTCCCA-3′
	Reverse	5′-CCACTGAGGCTCCTGATTCT-3′
hsa_circ_0007976	Forward	5′-TGAGAGAAATGCTTACACACAGA-3′
	Reverse	5′-ACAAAACCATCCAAGGCTTTCA-3′
hsa_circ_0001180	Forward	5′-AAGATGTGCTGTGCGATGTC-3′
	Reverse	5′-TGTCGGGAAGTTCAGTGGAA-3′
hsa_circ_0014478	Forward	5′-CTGCACCACTCAGCTCAAAG-3′
	Reverse	5′-GCAGCAGTTCTCCAAAGTGT-3′
hsa_circ_0025215	Forward	5′-AGGTTACAGATGTCAGAGCGT-3′
	Reverse	5′-GGGAATTCGGGCAATGGTAG-3′
hsa_circ_0025034	Forward	5′-GGTACCTATCCAGTTCCCGG-3′
	Reverse	5′-TGGGGTGAATGGTCCAGAAG-3′
hsa_circ_0016712	Forward	5′-CAAGGAGGATGCCATTGAGC-3′
	Reverse	5′-TCCTCAGACACAACTCGGAT-3′
hsa_circ_0016456	Forward	5′-CAAGAGGCAAAGATCCAGTGG-3′
	Reverse	5′-CTTGCAGGCGTAGTTCCAAC-3′
hsa_circ_0001791	Forward	5′-CAGTGCACAGCTAGTTCTTATG-3′
	Reverse	5′-CAGTCCAACTAGCACAATTCG-3′
U6	Forward	5′-ACGCAAATTCGTGAAGCGTT-3′
	Reverse	Uni-miR qPCR primer (Takara, Dalian, China)
GAPDH	Forward	5′-GCACCGTCAAGGCTGAGAAC-3′
	Reverse	5′-TGGTGAAGACGCCAGTGGA-3′

### Cell transfection

ShRNAs specifically targeting circ-ADAM9 and a non-targeting control shRNA were designed and synthesized at GeneChem. The shRNA nucleotide sequences were as follows: sh-circ-ADAM9–1: 5′-ACAGCTAGTTCTTATGACA-3′, sh-circ-ADAM9–2: 5′-GCTAGTTCTTATGACATGA-3′, sh-circ-ADAM9–3: 5′-CAGCTAGTTCTTATGA CAT-3′, nonsense shRNA: 5′-TTCTCCGAACGTGTCACGT-3′. The shRNA sequences were inserted into the GV493 lentiviral vector containing a gcGFP fluorescent marker and sequenced to confirm the correct identity of the shRNAs. For overexpression of circ-ADAM9, the circ-ADAM9 gene was cloned into the *Age*I/*Bam*HI site of the GV502 vector containing an EGFP fluorescent marker at GeneChem to construct the recombinant lentivirus, LV-circ-ADAM9. A negative control vector, EV-circ-ADAM9, was also constructed. The plasmid carrying the target gene and virus packaging helper plasmids (Helper 1.0, Helper 2.0) were purified and transfected into monolayer 293T cells cultured in DMEM for 48 h. The lentivirus was purified from the supernatant of the transfected 293T cells by centrifugation. The transfection efficiency was evaluated by fluorescence microscopy. The viral supernatant was transfected into human EPCs. Circ-ADAM9 levels were detected by RT-qPCR. Stable cell lines with knockdown and overexpression of circ-ADAM9 were established by puromycin (2 μg/mL) screening for further study in vitro and in vivo.

Mir-20a-5p mimics, inhibitor, and negative controls were purchased from GenePharma (Shanghai, China). The sequences were as follows: inhibitor: 5′-CUACCUGCACU AUGAGCACUUUG-3′; inhibitor negative controls: 5′-CAGUACUUUUGUGUAGU ACAA-3′; mimics: sense: 5′-CAAAGUGCUCAUAGUGCAGGUAG-3′, antisense: 5′-ACCUGC ACUAUGAGCACUUUGUU-3′; mimics negative controls: sense: 5′-UUC UCCGAACGUGUCACGUTT-3′, antisense: 5′-ACGUGACACGUUCGGAGAATT-3′. Silencer (si) RNAs for autophagy-related gene 7 (ATG7) and phosphate and tension homology deleted on chromosome 10 (PTEN), and control siRNA were synthesized at GenePharma. The siRNA nucleotide sequences were as follows: ATG7: sense: 5ʹ-CCAACACACUCGAGUCUUUTT-3ʹ, antisense: 5ʹ-AAAGACUCGAGUGUGUUG GTT-3ʹ, PTEN: sense: 5ʹ-CGCCAAAUUUAAUUGCAGATT-3ʹ, antisense: 5ʹ-UCUGCAAUUAAAUUUGGCGTT-3ʹ, negative controls: 5′-UUCUCCGAACGUGU CACGUTT-3′ and 5′-ACGUGACACGUUCGGAGAATT-3′. EPCs were transfected for 48 h with siRNAs, miRNA mimics and inhibitors, and negative controls using Lipofectamine 3000 (Invitrogen) according to manufacturer’s instructions.

### Analysis of cell autophagy and apoptosis

Cells were transfected with mir-20a-5p inhibitor, mir-20a-5p mimic, sh-circ-ADAM9, or LV-circ-ADAM9 for 24 h. Autophagy was evaluated by western blot analysis of LC3B-I, LC3B-II, and SQSTM1/p62. Autophagic flux was measured using a Premo Autophagy Tandem Sensor RFP-GFP-LC3 kit (P36239; Life Technologies) according to the manufacturer′s instructions. Fluorescence images were acquired after 48 h of incubation, using fluorescence microscope (Carl Zeiss, Jena, Germany).

Apoptosis was explored by western blot analysis of cleaved-CASP3, BAX, and BCL-2. Apoptotic EPCs were detected by flow cytometry. Annexin V^+^/PI^+^ and Annexin V^+^/PI^–^ cells (quadrants 2 and 3) were defined as apoptotic cells. In addition, apoptotic EPCs were detected using an In Situ Cell Detection Kit (11767291910; Roche Applied Science) according to the manufacturer′s instructions for terminal deoxynucleotidyl transferase dUTP nick-end labeling (TUNEL).

### Tube formation assay

A tube formation assay was used to evaluate the formation of capillary-like structures in vitro^47^. 96-well plates were coated with Matrigel (50 μL per well) at 37 °C for 30 min. EPCs (2 × 10^4^ cells per well) were seeded onto the Matrigel and cultured at 37 °C in the presence of 5% CO_2_. Tube formation was quantified after 6 h, using an inverted microscope (Olympus, Tokyo, Japan).

### Western blotting

Cells and tissues were lysed using a lysis buffer (9803S; Cell Signaling Technology, Shanghai, China) with 100 nM OmniPur Phenylmethyl Sulfonyl Fluoride (329–986; Calbiochem) and 1:100 Halt Phosphatase Inhibitor Cocktail (78420; ThermoScientific). Protein concentrations were determined using a bicinchoninic acid protein assay kit (P0011; Beyotime). The proteins were resolved in 6–15% polyacrylamide gels and transferred to polyvinylidene difluoride membranes (IPFL00010; Millipore). The membranes were incubated with primary antibodies at 4 °C overnight. Then, the membranes were washed with TBST (0.1% Tween-20 in TBS) three times and incubated with horseradish peroxidase-conjugated secondary antibodies (goat anti-rabbit: 111–035–003, goat anti-mouse: 115–035–003; Jackson ImmunoResearch) at 37 °C for 2 h. The membranes were exposed to X-ray films or imaged using a ChemiDoc MP Imaging System (Bio-Rad). Band intensities were determined using ImageJ v1.43 (NIH, Bethesda, MD, USA). Antibodies used in this study were: anti-cleaved-CASP3 (9664; Cell Signaling Technology), anti-BCL-2 (12789–1-AP; Proteintech); anti-BAX (50599–2-Ig; Proteintech), anti-LC3B (2775S; Cell Signaling Technology), anti-SQSTM1/p62 (5114; Cell Signaling Technology), anti-VEGF (sc-7269; Santa Cruz Biotechnology), anti-PTEN (9552; Cell Signaling Technology), anti-ATG7 (8558; Cell Signaling Technology), anti-p-AKT (4060; Cell Signaling Technology), anti-t-AKT (4691; Cell Signaling Technology), anti-p-mTOR (5536; Cell Signaling Technology), anti-t-mTOR (2972; Cell Signaling Technology), and anti-actin beta (A-5441; Sigma-Aldrich).

### Luciferase reporter assay

The 3′-UTRs of *ATG7* and *PTEN* containing wild-type or mutant mir-20a-5p were inserted into the pmiR-RB-REPORT vector (RiboBio, Guangzhou, China), and the circ-ADAM9 sequence containing wild-type or mutant mir-20a-5p was inserted into the psiCHECK2 vector (Promega, Madison, WI, USA) to construct dual-luciferase (firefly and Renilla) reporter plasmids. Cells were cotransfected with the wild-type or mutant luciferase vectors and mir-20a-5p mimic or negative control for 48 h. Firefly and Renilla luciferase activities were measured using a dual-luciferase system (Promega) according to manufacturer′s instructions. Firefly luciferase activity was normalized to that of Renilla luciferase.

### RNA-binding protein immunoprecipitation (RIP) assay

RIP assays were conducted using the Magna RIP™ RNA-Binding Protein Immunoprecipitation Kit (Millipore, Bedford, MA, USA). EPCs were incubated with RIP buffer containing magnetic beads conjugated with AGO2 antibody or negative control IgG. The retrieved RNAs were detected by RT-qPCR.

### Diabetic mouse hind limb ischemia model and EPC transplantation

Nude mice (6 weeks of age, male) were purchased from Shanghai Laboratory Animal Center (Shanghai, China) and were placed in a miniature isolator under a 12/12-h light/dark cycle for 4 weeks. Diabetes was induced by intraperitoneal injection of streptozotocin (Sangon Biotech, Shanghai, China). The mice received an injection of streptozotocin (50 mg/kg) or citrate buffer (4.92 mol/mL sodium citrate, pH 4.2–4.5) for 5 days consecutively. Blood glucose levels were measured on days 7, 14, and 21 after injection. Mice with blood glucose levels >12.0 mmol/L were considered diabetic and were used in the experiments.

Four weeks after diabetes induction, unilateral posterior limb artery disconnection was performed in all non-diabetic (*n* = 12) and diabetic mice (*n* = 24) as described previously^48^ to generate hind limb ischemia. The diabetic mice were randomly divided into two treatment groups (*n* = 12 in each group): mice in one group were injected with EPCs transfected with sh-circ-ADAM9 and mice in the other group were injected with EPCs transfected with negative control (sh-NC). Seventy-two hours after the operation, 200 mL of medium containing 1 × 10^5^ EPCs was injected intramuscularly in the ischemic limb at six sites distal to the vascular occlusion site^49^. A laser Doppler perfusion imaging system (Moor Instruments, Devonshire, UK) was used to monitor blood perfusion pre- and post-surgery, and on the 4th, 7th, and 14th day post-surgery. The ratio of perfusion in the ischemic limbs to that in the non-ischemic limbs was calculated. The investigator was blinded to the group allocation during the experiments.

The Institutional Animal Care and Use Committee of Shanghai Jiaotong University School of Medicine approved all of the above procedures and protocols, which were carried out in accordance with established International Guiding Principles for Animal Research.

### Immunofluorescence staining

Mouse tissue samples collected on day 7 after EPC injection were subjected to immunofluorescence staining. Tissue sections were fixed in 4% paraformaldehyde, incubated in blocking buffer (1% BSA and 0.3% Triton X-100 in PBS) for 2 h, and washed three times in Pblec buffer (1% Triton X-100, 1 mM CaCl_2_, 1 mM MgCl_2_, and 1 mM MnCl_2_ in PBS, pH 6.8). Capillaries in the ischemic muscles were visualized by anti-CD31 immunostaining. Samples were incubated with a FITC-conjugated anti-CD31 antibody (dilution 1:100; BD Pharmingen, San Diego, CA, USA) diluted in Pblec buffer at 4 °C overnight, under gentle rocking. Cells were observed in five random fields under a fluorescence microscope.

### Statistical analysis

Experimental data are reported as the mean ± SEM from at least three independent experiments and were analyzed using SPSS 17.0 (SPSS, Chicago, IL, USA). Means were compared using unpaired two-tailed *t*-tests in GraphPad Prism 5. *P*-value < 0.05 was considered statistically significant.

## Supplementary information

Supplementary figure legends

Supplementary figure S1

Supplementary figure S2

Supplementary figure S3

Supplementary figure S4
