# Supplementary material for: Circ-ADAM9 targeting PTEN and ATG7 promotes autophagy and apoptosis of diabetic endothelial progenitor cells by sponging mir-20a-5p
Source: Cell Death Dis. 2020 Jul 13;11(7):526. doi: 10.1038/s41419-020-02745-x (PMC7359341; doi:10.1038/s41419-020-02745-x)
Supplement: Supplementary file 1 — Supplementary figure legends [file 41419_2020_2745_MOESM1_ESM.docx]

**Supplementary figure legends**

**Fig. S1. Identification of human umbilical vein blood-derived EPCs.** (a) Immunostaining showed that adherent cells were positive for CD31 and CD34. Scale bar: 50 μm. (b) EPCs formed capillary-like networks when seeded onto Matrigel surfaces. Scale bar: 200 μm. (c) Flow-cytometric analysis showed that adherent cells were positive for CD31, CD34, CD133, and VEGFR-2, indicating the characteristics of EPCs. The control group showed blank curve and the test sample showed solid grey curve. (d) EPCs derived from human umbilical vein blood were further confirmed by their ability to bind FITC-UEA-1 and to ingest Dil-acetylated low-density lipoprotein. Scale bar: 200 μm.

**Fig. S2 Screening of circ-ADAM9.** (a) Schematic illustration of target circRNAs of mir-20a-5p predicted by each of miRanda, TargetScan, and RNAhybrid. (b) EPCs were incubated in medium containing 30 mM glucose or 30 mM mannitol for the indicated periods. Treatment with mannitol was used as an osmolar control treatment. Expressions of the 15 candidate circRNAs was determined by RT-qPCR. **P* < 0.05; ***P* < 0.01; n = 3

**Fig. S3. PTEN and ATG7 expression is downregulated in EPCs.** (a) Western blot analysis of PTEN in EPCs. (b) Western blot analysis of ATG7 in EPCs. **P* < 0.05; ***P* < 0.01; n = 3

**Fig. S4. Inhibition of EPCs autophagy by 3-MA under high glucose condition.** Western blot analysis of LC3B and P62 in EPCs under high glucose condition. **P* < 0.05; ***P* < 0.01; n = 3
