# Supplementary figures and images for: Circ-ADAM9 targeting PTEN and ATG7 promotes autophagy and apoptosis of diabetic endothelial progenitor cells by sponging mir-20a-5p
Source: Cell Death Dis. 2020 Jul 13;11(7):526. doi: 10.1038/s41419-020-02745-x (PMC7359341; doi:10.1038/s41419-020-02745-x)

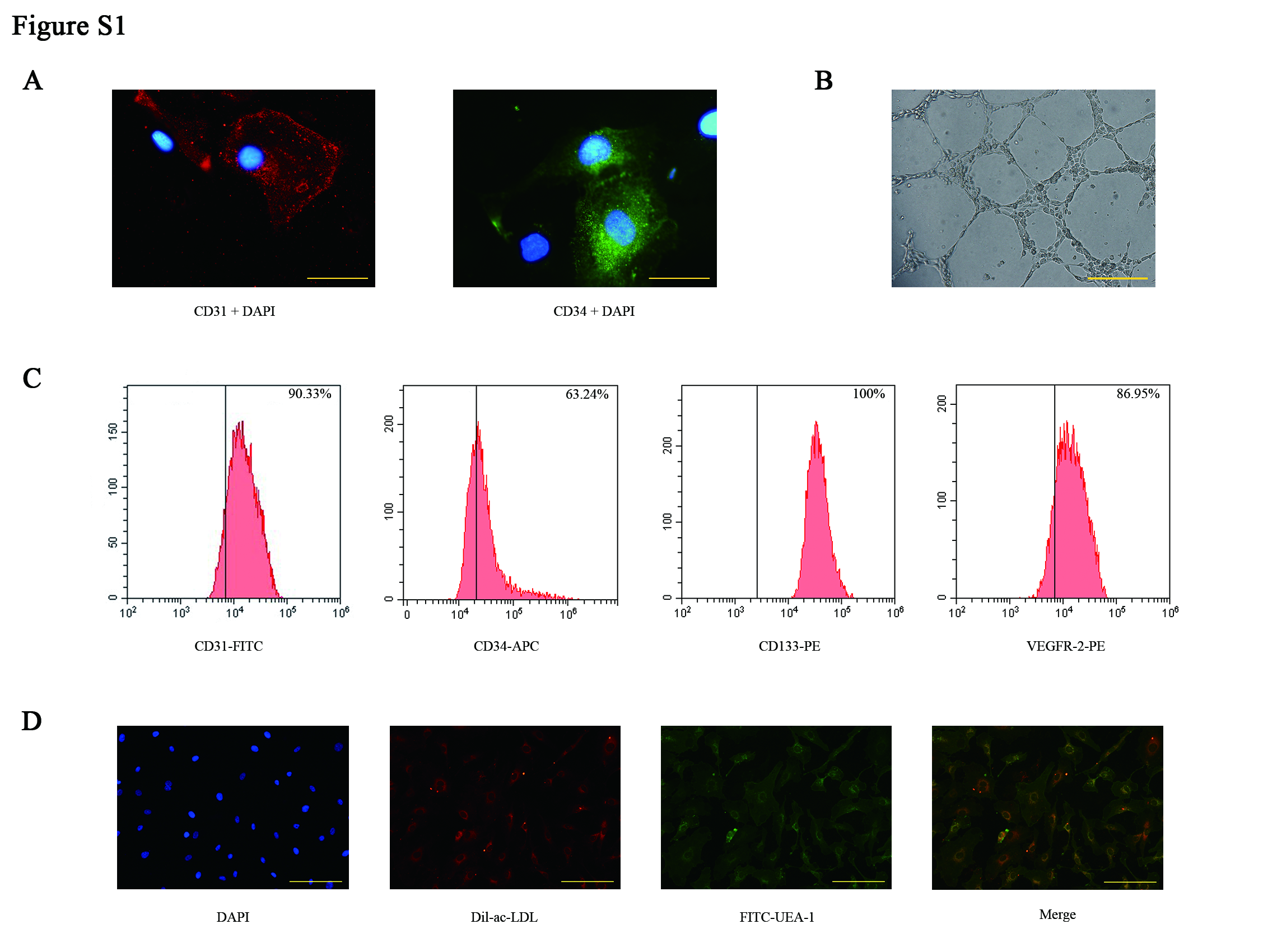

Supplement: Supplementary file 2 — Supplementary figure S1 [file 41419_2020_2745_MOESM2_ESM.tif]

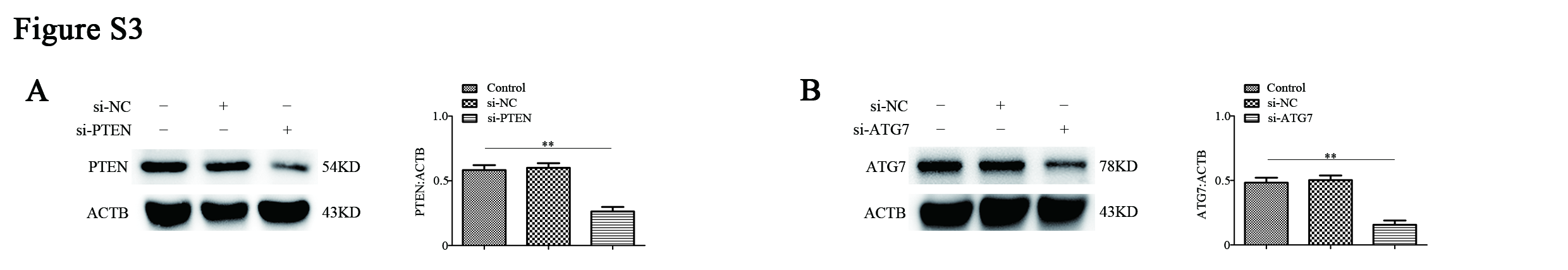

Supplement: Supplementary file 4 — Supplementary figure S3 [file 41419_2020_2745_MOESM4_ESM.tif]

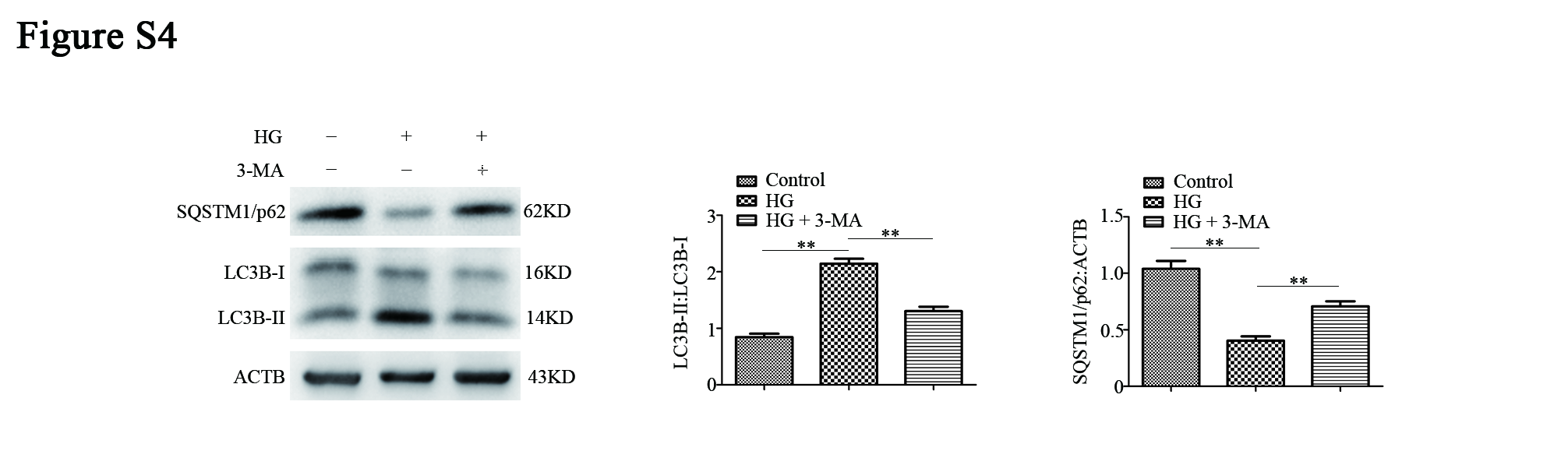

Supplement: Supplementary file 5 — Supplementary figure S4 [file 41419_2020_2745_MOESM5_ESM.tif]
